# Development of a spore-based mucosal vaccine against the bovine respiratory pathogen *Mannheimia haemolytica*

**DOI:** 10.1038/s41598-023-29732-4

**Published:** 2023-08-10

**Authors:** Muhammed Salah Uddin, Jose Ortiz Guluarte, D. Wade Abbott, G. Douglas Inglis, Le Luo Guan, Trevor W. Alexander

**Affiliations:** 1 Lethbridge Research and Development Centre, Agriculture and Agri-Food Canada, 5403 1st Avenue South, Lethbridge, AB T1J 4B1 Canada; 2 Department of Agricultural, Food and Nutritional Science, University of Alberta, Edmonton, AB T6G 2P5 Canada

**Keywords:** Protein vaccines, Vaccines

## Abstract

Bovine respiratory disease (BRD) is a significant health issue in the North American feedlot industry, causing substantial financial losses due to morbidity and mortality. A lack of effective vaccines against BRD pathogens has resulted in antibiotics primarily being used for BRD prevention. The aim of this study was to develop a mucosal vaccine against the BRD pathogen, *Mannheimia haemolytica*, using *Bacillus subtilis* spores as an adjuvant. A chimeric protein (MhCP) containing a tandem repeat of neutralizing epitopes from *M. haemolytica* leukotoxin A (NLKT) and outer membrane protein PlpE was expressed to produce antigen for adsorption to *B. subtilis* spores. Adsorption was optimized by comparing varying amounts of antigen and spores, as well as different buffer pH and reaction temperatures. Using the optimal adsorption parameters, spore-bound antigen (Spore-MhCP) was prepared and administered to mice via two mucosal routes (intranasal and intragastric), while intramuscular administration of free MhCP and unvaccinated mice were used as positive and negative control treatments, respectively. Intramuscular administration of MhCP elicited the strongest serum IgG response. However, intranasal immunization of Spore-MhCP generated the best secretory IgA-specific response against both PlpE and NLKT in all samples evaluated (bronchoalveolar lavage, saliva, and feces). Since proliferation of *M. haemolytica* in the respiratory tract is a prerequisite to lung infection, this spore-based vaccine may offer protection in cattle by limiting colonization and subsequent infection, and Spore-MhCP warrants further evaluation in cattle as a mucosal vaccine against *M. haemolytica.*

## Introduction

Bovine respiratory disease (BRD), also known as shipping fever, continues to be a challenging health issue resulting in significant economic losses due to morbidity and mortality in North American cattle^1–3^. Treatment and control of BRD in the beef sector are aimed mainly at bacterial pathogens, through the use of antibiotics and vaccination programs. However, there are public and scientific concerns regarding overuse of antibiotics in livestock production^4^, and current vaccines do not afford complete protection against disease^5^. Novel methods to mitigate BRD-related pathogenic bacteria are therefore greatly needed. *Mannheimia haemolytica* is systematically detected in BRD cases and it is the predominant bacterial agent associated with the disease^6,7^. Because of its recognized importance, development of vaccines against *M. haemolytica* in the form of bacterins, cell culture supernatants, and extracted antigens have been produced^6^, but have yielded variable results in controlled trials, and limited efficacy in production settings^8^. Timing of vaccination (eg. pre- versus post- feedlot arrival), antigen combinations (eg. bacterial and viral antigens mixed), stress factors, and levels of maternal antibodies may affect vaccine efficacy^9,10^. While this tapered potency may partly be due to vaccines offering narrow protection across strains of the bacterium, the systemic immunity targeted by intramuscular injection^11,12^ may also be a factor. Mucosal immunization can be advantageous over parenteral delivery since it can induce both mucosal and systemic immunity, providing protection at the site of infection^13^.

The delivery method used plays a key role in the successful transition of chimeric protein into an effective vaccine^14,15^. A poor delivery system can lead to loss of biological activity, which often negates antigen efficacy. To date, a wide range of delivery systems, ranging from live microorganisms to nanoparticles, have been proposed as alternative vaccine vehicles^15^. Probiotic bacteria have increasingly been studied to deliver antigens and elicit mucosal responses because of their natural ability to interact with animal mucosa and regulate immune responses. Previously, a non-transgenic application of *Bacillus subtilis* endospores (referred to as spores from here on) that allowed binding of antigens in their native state for presentation to mucosa was evaluated^16^. This technology was shown to stimulate mucosal immunity via oral and intranasal administration, providing protection against bacterial and viral infection in mice^16,17^. There are potential commercial benefits of using this technology for vaccinating cattle against BRD pathogens. Production of *Bacillus subtilis* is well established and has low cost inputs, and *B. subtilis* is used commercially in food/feed products for human beings, poultry, cattle, swine, and fish, as it is recognized as a probiotic that has generally regarded as safe (GRAS) status^18,19^. Thus, a low-cost and GRAS vaccine component with a history as an adjuvant would facilitate industry adoption. In addition, oral administration of the vaccine would allow for large-scale administration. These qualities are especially important as livestock management strategies, including vaccination, are cost and ease-of-use dependent. To date, *B. subtilis* spores have not been tested as vaccine delivery systems for cattle pathogens.

Immunity against *M. haemolytica* requires generation of antibodies against the virulence factor, leukotoxin (LKT), and to cell surface antigens^20,21^. Multiple studies have demonstrated that outer membrane proteins are immunologically important surface antigens^22–25^. Among them, vaccination of cattle with the 45 kDa, surface-exposed, outer membrane lipoprotein, PlpE, was shown to generate a higher degree of resistance to disease incited by *M. haemolytica*^22,23,26^. In the current study, we used a *M. haemolytica* chimeric protein CTB-PlpE-NLKT-PlpE-NLKT (MhCP), which is composed of truncated Cholera Toxin B subunit (CTB), two copies of the immune-dominant epitope (R2) region of *M. haemolytica* PlpE, and two copies of the neutralizing epitope of LKT (NLKT)^27^. The purified MhCP was then adsorbed to *B. subtilis* spores, and the resultant spore-based vaccine termed “Spore-MhCP” was evaluated in mice for the induction of immunogenicity. The objectives of this study were to first, develop a spore-based vaccine targeting *M. haemolytica* and second, evaluate the immunogenicity of this vaccine after delivery via mucosal (intranasal and intragastric) routes.

## Results

### Adsorption of MhCP to spores

**Figure 1:**
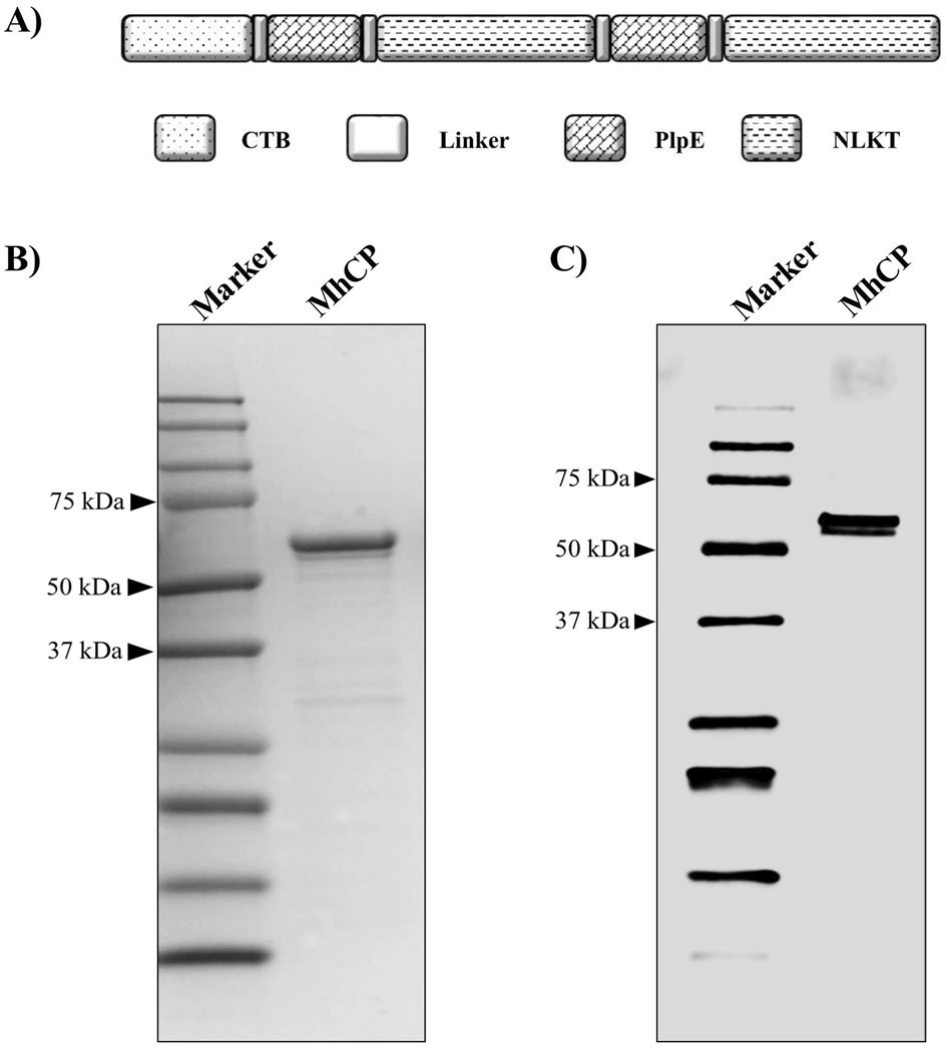
Construction and purification of chimeric protein MhCP. (**A**) Modular structure of chimeric protein MhCP (CTB + PlpE + NLKT + PlpE + NLKT). MhCP contains truncated cholera toxin subunit B (CTB), immunodominant surface epitope (R2) of *M. haemolytica* outer membrane lipoprotein PlpE, and the neutralizing epitope of leukotoxin (NLKT). MhCP was synthesized and subcloned into the bacterial expression vector, pET28a. The purified plasmids were transformed into *E. coli* BL21 (DE3) protein expression cells. The recombinant chimeric protein MhCP was purified using a NTA-nickel column. (**B**) Purified recombinant chimeric protein MhCP demonstrated by sodium dodecyl sulfate–polyacrylamide gel electrophoresis (SDS-PAGE), stained with Coomassie Brilliant Blue. (**C**) Western blots were used to confirm the identity of the MhCP protein by using anti-His antibodies.

**Figure 2:**
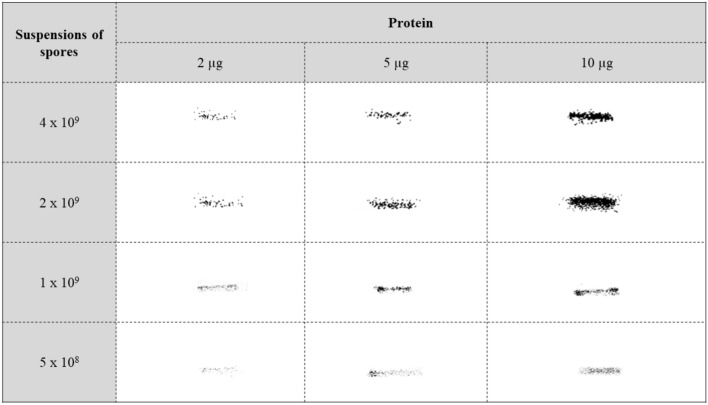
Optimization of spore-to-antigen ratio. Chimeric protein, MhCP, of varying amounts (2 µg, 5 µg, 10 µg) were mixed with a range of *Bacillus subtilis* spores (5 × 10^8^ to 4 × 10^9^) in phosphate buffered saline (PBS), incubated for 1 h, washed, and resuspended in PBS. The supernatant was removed, the pellet containing spore coat antigens was washed and extracted by incubating in extraction buffer for 30 min at 65 °C. Quantities of MhCP were determined by Western blotting. Full-length blots, used to create this figure, are available in the Supplemental Fig. S2.

**Figure 3:**
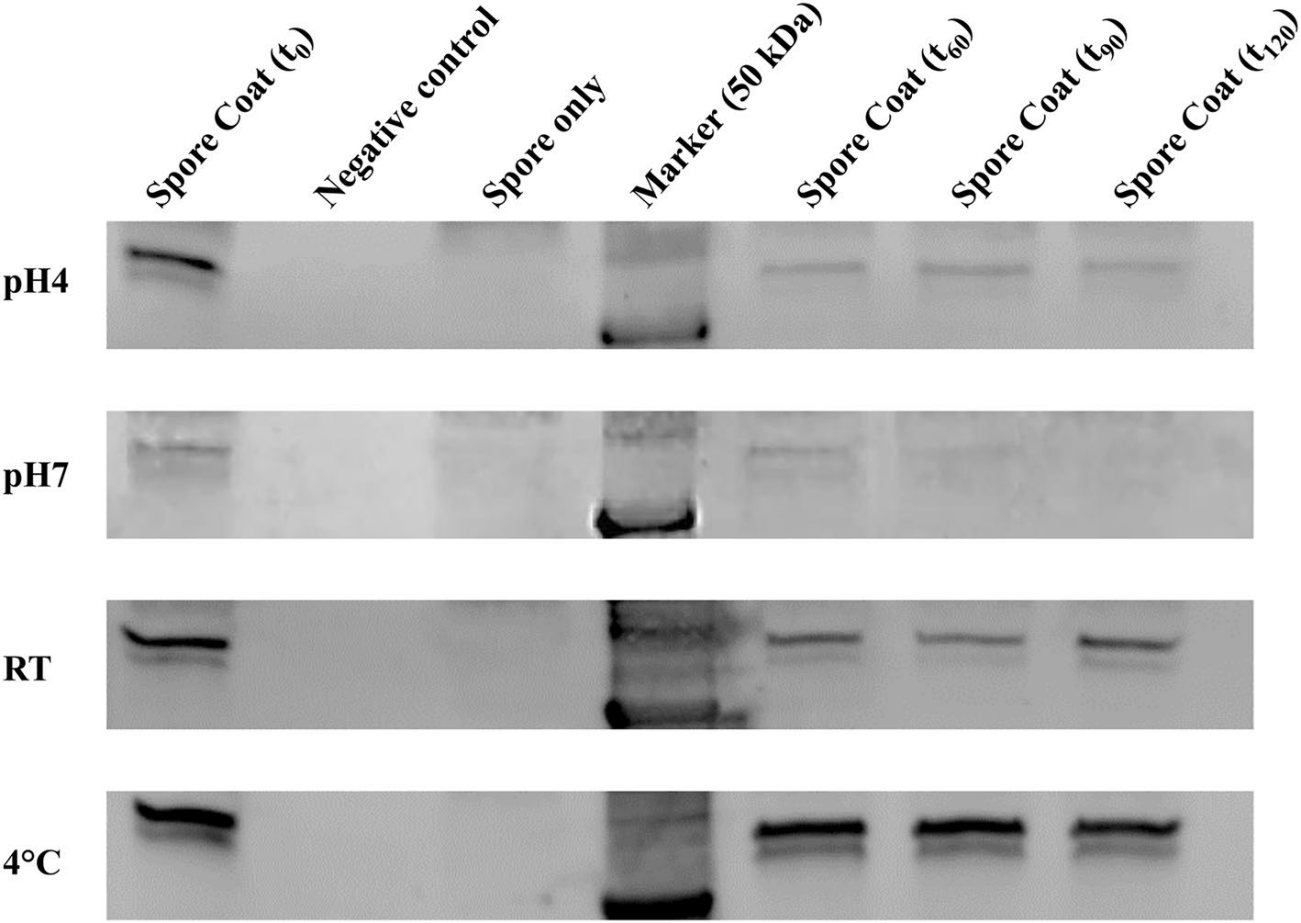
Adsorption of chimeric protein MhCP to *Bacillus subtilis* spores. MhCP adsorbtion to spores was evaluated at different pHs and temperatures. 10 µg of MhCP was mixed with 2 × 10^9^ spores in phosphate buffered saline (PBS) at pH 4 and PBS at pH 7, incubated 1 h at room temperature (RT). The binding mixture was centrifuged, the supernatant discarded, and the pellet was washed two times with PBS. The washed pellet was next resuspended in 200 µl of PBS at their respective pH. At indicated time points (60, 90 and 120 min), the spore suspensions were centrifuged. Pellets were resuspended in 100 µl of spore coat extraction buffer, incubated at 65 °C for 30 min to remove spore coat proteins from the spores. Using a one in ten dilution of the extraction, western blotting of size-fractionated proteins was used for detection. Equivalent amounts of spores and protein were used for third and fourth panel (pH 4). After the initial 1 h incubation at RT, the binding mixture was incubated at 4 °C (third panel), whereas all reactions and incubations were performed at 4 °C (fourth Panel). The negative control lane displays the corresponding blot that underwent the entire process without addition of any spores or protein. The spore only lane displays the corresponding blot generated from spores that underwent the entire process without the addition of protein. Full-length blots, used to create this figure, are available in the Supplemental Fig. S3.

The modular structure of MhCP is shown in Fig. 1A. The size, purity, and integrity of the recombinant protein MhCP was demonstrated by sodium dodecyl sulfate–polyacrylamide gel electrophoresis (SDS-PAGE; Fig. 1B) and western blotting (Fig. 1C). Results from the adsorption experiment revealed that the best binding condition was when 10 µg of antigen, MhCP, was mixed with 2 × 10^9^ spores, as indicated by the band intensity from western blotting (Fig. 2). Among the tested parameters, MhCP was efficiently adsorbed onto the spore coat at pH 4, whereas at pH 7, low levels of binding were observed (Fig. 3). The temperature also played a key role in binding, and the degree of binding was most stable at 4 °C (Fig. 3). Similar band intensity was observed by western blotting across the tested time points of 60 min, 90 min and 120 min respectively, highlighting stable binding of the Spore-MhCP complex (Fig. 3).

### Spore-MhCP induced antigen-specific antibody production

**Figure 4:**
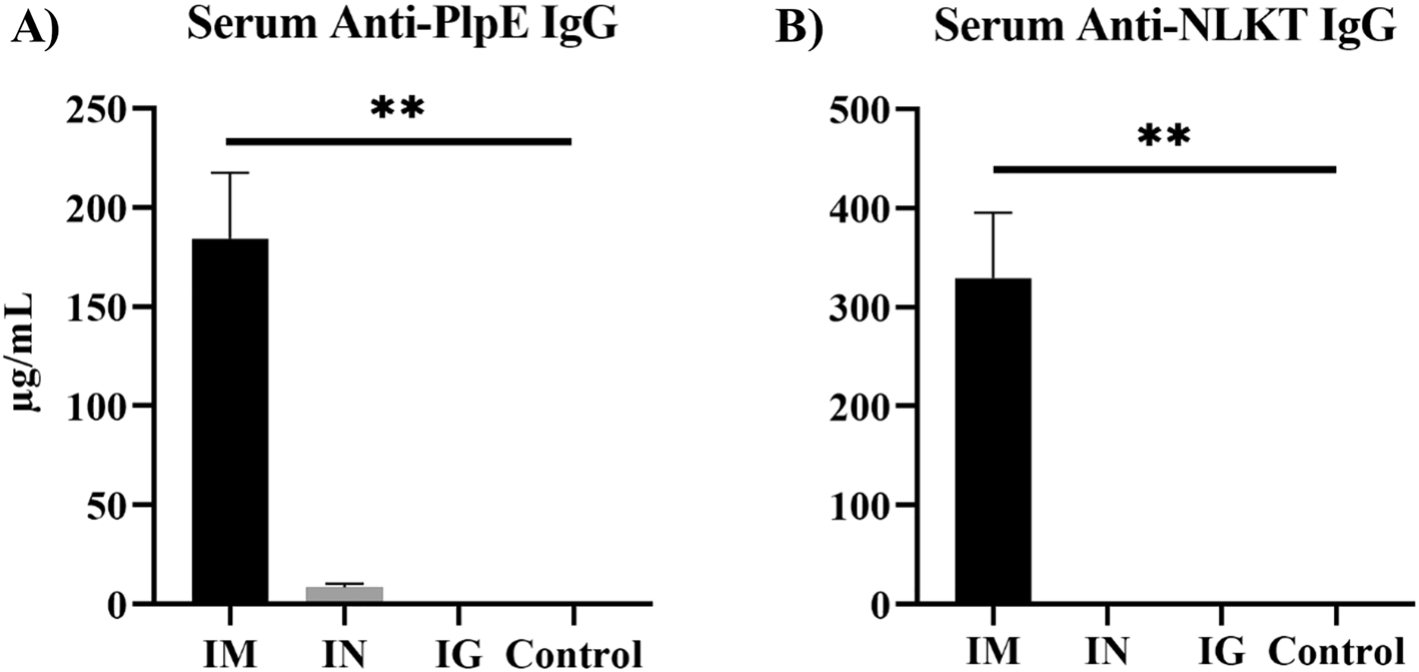
Antigen-specific serum IgG antibody responses measured by enzyme linked immunosorbent assay (ELISA) from blood collected on day 42. ELISA plates coated with either recombinant PlpE or NLKT were used to measure anti-PlpE and anti-NLKT antibodies in mice sera. Immune responses from four experimental groups: Intramuscular (IM), Intranasal (IN), Intragastric (IG) and Naïve/control mice (Control) were compared. (**A**) Levels of serum IgG specific to PlpE (**B**) Levels of serum IgG specific to NLKT. Results are expressed as mean ± SEM. Significance was tested against the control by Kruskal–Wallis test with post-hoc Dunn’s multiple comparison test, **p < 0.01.

**Figure 5:**
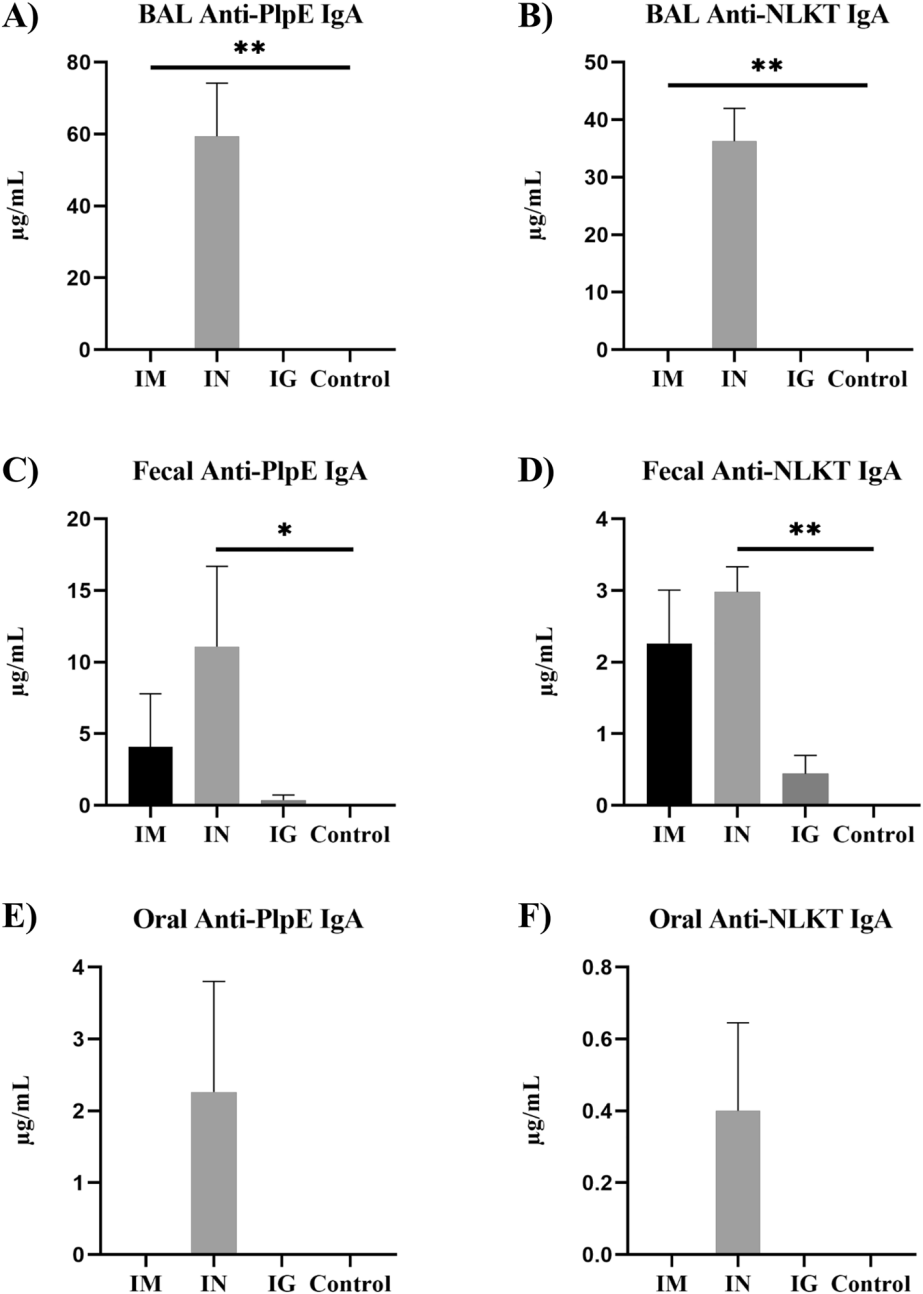
Enhanced mucosal immunity induced by Spore-MhCP. Mucosal immune responses measured by ELISA where plates were coated with either recombinant PlpE or NLKT to measure anti-PlpE IgA and anti-NLKT IgA antibodies in bronchoalveolar lavage (BAL), fecal and oral swab samples obtained from mice on day 42. Immune responses from the four experimental treatments, intramuscular (IM), intranasal (IN), intragastric (IG) and naïve/control mice (Control) were compared. Results are expressed as mean ± SEM. Significance was tested against the control by Kruskal–Wallis test with post-hoc Dunn’s multiple comparison test, **p < 0.01 and *p < 0.05.

Immunization of mice with both free MhCP and Spore-MhCP stimulated anti-PlpE and anti-NLKT antibodies indicating that the chimeric antigen MhCP that was used was immunogenic, and retained immunogenicity after spore adsorption (Figs. 4, 5). On both days 21 (Supplemental Fig. S1) and 42 (Fig. 4), intramuscular treatment mice exhibited a greater serum IgG immune response as compared to other treatment mice for both the PlpE (*P* < 0.05) and NLKT (*P* < 0.05). The IgG immune response increased several fold in intramuscular treatment mice by day 42. However, the intranasal group showed numerically higher serum anti-PlpE antibodies as compared to intragastric and control treatment mice at both time points (*P* > 0.05). The control and intragastric treatment mice did not show any detectable serum antibody production (detection limit ≥ 7.8 ng/ml) on days 21 or 42.

On day 21, none of the mucosal samples showed any detectable IgA immune responses (detection limit ≥ 7.8 ng/ml; data not shown). On day 42, intranasal vaccination with Spore-MhCP resulted in specific secretory anti-PlpE and anti-NLKT IgA in bronchoalveolar lavage (BAL) samples (Fig. 5A,B). None of the other treatment mice exhibited detectable levels of secretory antibody in BAL fluid. The intranasal treatment mice also had greater amounts of anti-PlpE IgA in both fecal (Fig. 5C) and oral swab (Fig. 5E) samples as compared to intragastric and control treatment mice. Intranasal treatment mice also exhibited greater amounts of anti-NLKT IgA in feces as compared to intragastric and control treatment mice (*P* < 0.05; Fig. 5D). The results showed that although PlpE and NLKT were delivered together as a chimera, differential immune responses were observed for each antigen. Anti-PlpE IgA and anti-NLKT IgA were also observed in feces from mice that were vaccinated intramuscularly with free MhCP (Fig. 5C,D).

### Bactericidal activity of mice sera immunized with MhCP

**Figure 6:**
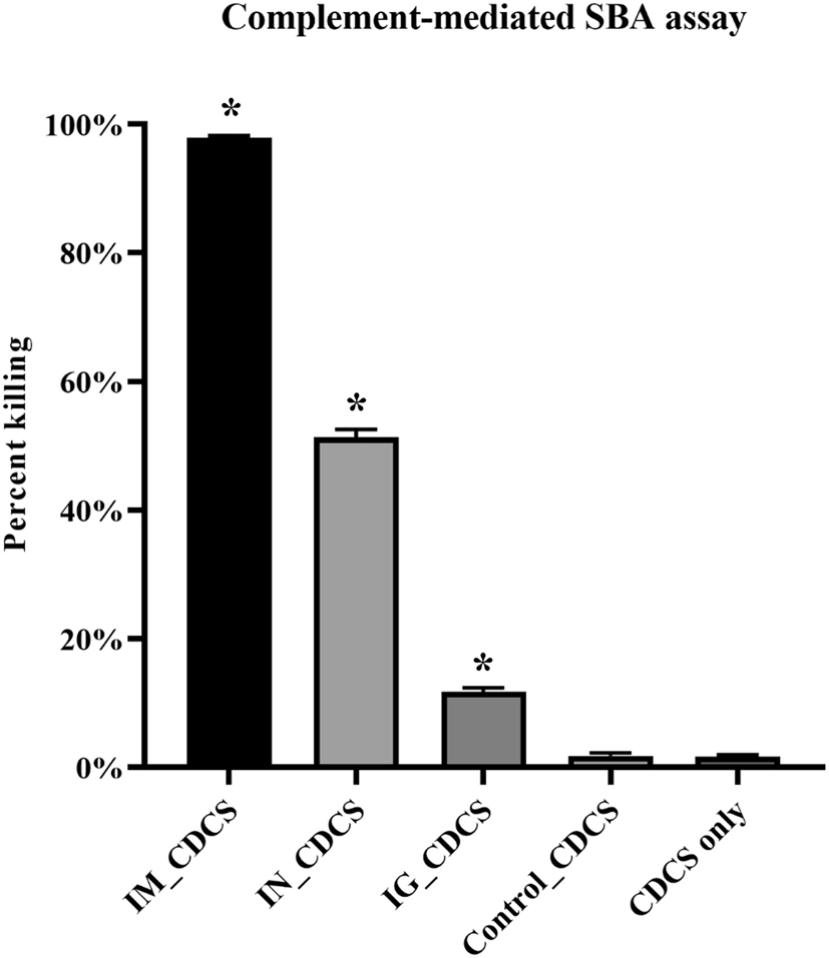
Complement-mediated serum bactericidal activity. Antibodies in mice sera collected on day 42 were evaluated for their killing activity against *M. haemolytica.* Bactericidal activity of mice sera collected from the four experimental treatments, intramuscular (IM), intranasal (IN), intragastric (IG) and naïve/control mice (Control) were compared. All mice sera analyzed were undiluted. Colostrum-deprived calf serum (CDCS) collected from a newborn calf was used as external source of complement. Only complement source (CDCS only) without serum/antibodies served as negative control. Results are expressed as mean ± SEM. Significance was tested against the control by one-way ANOVA with Tukey’s multiple comparison test, *p < 0.05.

The ability of vaccine-induced antibodies to mediate complement-dependent killing of *M. haemolytica* was tested using a serum bactericidal activity (SBA) assay. Sera from the treatment groups were evaluated for their bactericidal activity in the presence of complement. The newborn calf serum used as a complement source had minimal bactericidal activity, which was also the case for the control treatment (Fig. 6). Sera from the intramuscular treatment mice demonstrated a high bactericidal activity (97.9%) compared to other treatment mice (*P* < 0.05; Fig. 6). The intranasal and intragastric group also showed higher bactericidal activity (51.3% and 11.8%, respectively) compared to control treatment mice (*P* < 0.05; Fig. 6).

## Discussion

To determine the optimal adsorption parameters, we compared the binding of spores and chimeric protein under varying amounts of each, as well as different buffer pH and reaction temperatures. Studies have shown that proteins can readily bind to negatively charged bacterial spores when the pH of the aqueous phase falls below the isoelectric point (pI) of the respective protein^16,17^. Since the pI of the protein, MhCP, was 9.1, we conducted binding experiments at pH 4 and pH 7. MhCP was most efficiently adsorbed onto the spore coat at pH 4, whereas at pH 7, lower levels of binding were observed. This finding is similar to other studies that have reported that the degree of protein adsorption to spores depended on the solution pH as well as the structure of the protein^16,28^. It is thought that electrostatic binding and hydrophobic effects are key ways in which proteins interact with spores, resulting in their adsorption^29^. In the current study, we showed that the temperature was also an important factor in adsorption, with the highest degree of adsorption occurring at 4 °C. It is important to maintain the native conformation of the immunogen to generate protective immunity^30^, and studies have reported that protein can maintain native conformation when bound to spores^16^. The small size of spores (approximately 1 µm in diameter) has its own advantages since it aligns them with particulate adjuvants where an antigen adsorbed on a surface of spores mimics a potential pathogen thereby facilitating antigen presentation and induction of an immune response^31,32^.

In this current study, we developed a spore based vaccine, Spore-MhCP, targeting chimeric protein MhCP of *M. haemolytica*. Chimeric proteins are versatile in nature and can deliver multiple antigens to the immune system which makes them ideal candidates for vaccine development^33,34^. The chimeric protein, MhCP encoded NLKT, which is the predominant virulence factor of *M. haemolytica,* and an epitope (R2) of PlpE, which is an outer membrane lipoprotein that is conserved in key *M. haemolytica* serotypes^35^. Although MhCP was developed as chimeric protein, both antigens (NLKT and PlpE) resulted in seroconversion individually. A previous study by Ayalew et al. (2008) showed that the components of MhCP are antigenic^27^, which is also evident from the immune responses generated against NLKT and PlpE following intramuscular immunization of mice with free MhCP in the current study. Ayalew et al. (2008) subcutaneously immunized mice with 25–75 μg of a similar chimeric protein (without CTB appended to it), and they reported that increasing doses of chimeric protein were associated with increasing anti-LKT antibodies^27^. In the current study, anti-NLKT and anti-PlpE antibodies were induced with 5 μg of MhCP after intramuscular administration, perhaps indicating that the CTB in our MhCP protein structure enhanced immunogenicity.

To investigate the effectiveness of the vaccine in vivo, three different routes of immunization were compared using a mice model. It was demonstrated that the antigen, MhCP, bound to spores in a manner that retained immunogenicity. Intranasal immunization of mice with Spore-MhCP stimulated mucosal immunity in the form of secretory IgA against *M. haemolytica* NLKT and PlpE. Therefore, *B. subtilis* spores could serve as an effective adjuvant for a mucosal vaccine. While intranasal Spore-MhCP did not elicit as strong an IgG response as did intramuscularly administered free MhCP, it resulted in significantly higher specific secretory IgA at all mucosal sites evaluated. Thus, intranasal Spore-MhCP immunization resulted in both mucosal and systemic immune response. This suggests that as a vaccine, improved protection may be conferred against *M. haemolytica* colonization in the upper and lower respiratory tract, including in the lungs of cattle.

Serum bactericidal activity evaluates the complement-mediated functional activity of vaccine-induced antibodies, and their ability to kill the targeted bacteria of interest^36^. Vaccine-induced antibodies from the intramuscular treatment mice exhibited almost complete killing of *M. haemolytica* in the complement-dependent SBA assay. This result is consistent with bactericidal activity observed after IM vaccination of mice with a similar construct, reported previously^27^. While sera from the intranasal treatment mice had a lower value of bactericidal activity compared to intramuscular mice, this was likely due to reduced IgG levels resulting from intranasal immunization^37^. Surprisingly, although intragastric mice failed to showcase any detectable range of serum IgG, sera from this group had bactericidal activity. This highlights an important fact that the spore-bound MhCP antigen had the potential to stimulate functional antibodies against *M. haemolytica* whether it was administered in free form via intramuscular route or with spore-bound mucosal routes. Unfortunately, it was not possible to measure bactericidal activities of BAL samples due to the low volumes collected, but measuring this activity in future studies in cattle would provide important insight into whether the spore-bound vaccine could reduce colonization by *M. haemolytica* at mucosal surfaces.

Oral vaccines are appealing for the livestock industry because they can be easily administered to livestock animals without extensive expertise. Although intranasal Spore-MhCP resulted in both mucosal and systemic immunity, intragastric administration only generated a limited immune response in fecal samples. This was likely due in part to antigen dilution in the stomach, and degradation from a low gastric pH together with gastric and intestinal proteases^38^. Studies have reported induction of systemic and secretory antibodies after intragastric immunization of spore-based vaccines in mice against *Clostridium perfringens*^16^ and *Bacillus* anthracis^39^. However, recombinant *B. subtilis* was used for those studies, and it was engineered to express antigens on the spore surface that were fused to the outer coat protein, which may have provided superior protection. Successful oral immunization with spore-bound antigens is likely related to their stability on the spore surface and enhanced protection from proteolysis. Because spore coat composition is strain-specific, and variation in spore coat material may affect antigen adsorption and protection^40^, antigens with pI approaching 2–3 would be favoured to remain adhered and protected while transiting the low pH environment of monogastric stomachs. Alternatively, a fibre-rich diet fed to cattle would promote retained adsorption of proteins with higher pI, by promoting a ruminal pH greater than 5.8^41^. Although oral administration was unsuccessful to generate detectable immune responses in mice, evaluation of spore-based vaccines in ruminants is warranted given anatomical differences and rumination potentially resulting in stimulation of pharyngeal lymphoid tissue by antigens in feed^12^.

Mucosal vaccination of cattle may be superior to parenteral vaccination for prevention of respiratory disease, as intranasal replication of respiratory pathogens is the first phase in development of BRD^12,42,43^. Ayalew et al. (2009) intranasally vaccinated weaned beef calves with R2-NLKT-R2-NLKT chimeric protein in the presence and absence of native cholera toxin^44^. They showed that vaccination with the chimeric protein enhanced resistance against intrabronchial challenge with *M. haemolytica*, as well as stimulating antibody responses to the bacterium^44^. However, the antibody responses only significantly increased following bacterial challenge. Batra et al. (2017) recently developed a recombinant BHV-1-vectored vaccine expressing PlpE-LKT chimeric proteins, and they reported production of anti-LKT antibodies after intranasal vaccination of bighorn sheep^45^. However, the development of antibodies against surface antigen was inconsistent, and the vaccine failed to protect against *M. haemolytica* challenge^45^. Because intranasally delivered Spore-MhCP produced both mucosal and systemic immune responses in mice, we hypothesise that it may confer effective protection against *M. haemolytica* infection in cattle. Findings from the current study in mice indicate that further studies to determine the efficacy of the *B. subtilis* spore-based mucosal vaccine in cattle to manage BRD are warranted.

## Materials and methods

### Preparation of spores

Lyophilized *B. subtilis* (RK28 strain) spores were obtained from SporeGen Limited (Egham, Surrey, UK). Prior to use, lyophilized spores were washed sequentially with 1 M KCl, 0.5 M NaCl, and distilled water (three times) by mixing 0.1 g of spore suspension with 10 ml of each solvent, followed by centrifugation (5000×*g*; 15 min) and decanting of the supernatant. The washed spores were heat treated at 65 °C for 30 min to kill vegetative cells, and the suspension was diluted in a tenfold dilution series, 100 µl spread onto Luria Bertani (LB) agar, cultures were incubated overnight at 39 °C, and colonies were counted at the dilution yielding 30–300 colony forming units per dish. After enumeration, washed spores were diluted in water to a final density of 1.0 × 10^10^ *B. subtilis* spores/ml.

### Construction, expression and purification of chimeric protein

The construction of the *M. haemolytica* chimeric protein MhCP was performed as per the modular structure depicted at Fig. 1A. MhCP was comprised of truncated cholera toxin B subunit (CTB), and two copies each of the immunodominant surface epitope (R2) of *M. haemolytica* PlpE and the neutralizing epitope of leukotoxin (NLKT). The chimeric protein MhCP has a calculated molecular weight of 56.4 kDa and a pI of 9.1. A similar chimeric protein (R2-NLKT-R2-NLKT) was used previously by Ayalew and colleagues with the exception of CTB, which was not appended to their protein structure^27^. Ayalew et al., (2009) reported another chimeric protein (CTB-R2-NLKT) with appended CTB and it differs with the number of constructs used in this current study^44^. The PlpE and NLKT constructs of MhCP shares identical amino acid sequences to R2 and NLKT used previously^27,44^. Sequences corresponding to the chimeric protein, MhCP were commercially synthesized (BioBasic Inc., Markham, Canada), codon optimized for expression in *E. coli*, and cloned into the pET28a vector. The pET28a constructs were transformed into *E. coli* BL21 Star (DE3), and recombinant chimeric protein were expressed and purified as previously described^46^. Briefly, cells were grown at 37 °C to an optical density (600 nm) of 0.8–1.0 in LB broth containing kanamycin (50 µg/ml). Cultures were cooled to 16 °C, and gene expression was induced by addition of 0.4 mM isopropyl 1-thio-β-d-galactopyranoside (IPTG) with overnight incubation at 200 rpm. Overnight cultures were centrifuged at 6500×*g* for 10 min. Cells were resuspended in binding buffer (0.5 M NaCl, 20 mM Tris, pH 8.0), and lysed by sonication for 2 min of 1-s intervals of medium intensity sonic pulses (Fisherbrand™ Model 505 Sonic Dismembrator). The cell lysate was clarified by centrifugation at 17,500×*g* for 45 min, and the pellet was washed sequentially with dH_2_0, binding buffer with 0.5% (w/v) Triton X-100, and finally with binding buffer only. The washed pellet was resuspended in binding buffer with 6 M urea with vigorous stirring at ambient temperature. The solubilized pellet was clarified by centrifugation at 20,000×*g* for 45 min, and the supernatant containing the recombinant protein was passed through a 0.45-µm filter. The filtrate was then loaded onto a nickel nitrilotriacetic acid column, and purified by immobilized metal affinity chromatography. The recombinant protein was eluted via a stepwise gradient of imidazole (5, 10, 30, 100 and 500 mM) in binding buffer with 6 M urea. Fractions containing significant amounts of protein were pooled, the denaturant (6 M urea) was removed by step wise dialysis, and buffer exchanged into storage buffer (20 mM Tris–HCl, pH 8.0). Following buffer exchange, samples were concentrated using ultrafiltration cell (Amicon) with a molecular mass cut-off of 10 kDa. The identity, purity, and integrity of purified proteins were determined by SDS-PAGE and confirmed by western blotting using anti-His antibodies (ThermoFisher, Canada).

### Spore binding with the chimeric protein

To determine the optimal condition for antigen (MhCP) binding to spores, varying amounts of MhCP (2 µg, 5 µg, and 10 µg) and spores (5 × 10^8^ to 4 × 10^9^) at different buffer pH (4 and 7) and varied temperature (room temperature and 4 °C) were tested^16^. Subsequent to each of the conditions employed, the Spore-MhCP mixture was centrifuged, washed, and then analysed for adsorption of MhCP to spores. For all criteria tested, optimal conditions were considered those that resulted in the greatest amount of MhCP binding to the spore coat, as determined by western blotting. The optimized method for adsorbing protein MhCP to endospores, which was used for vaccine preparation in the mice study follows. An aliquot of *B. subtilis* spores containing 2 × 10^9^ spores were centrifuged (5000×*g*, 5 min, 4 °C), the supernatant was removed, and 10 µg of purified recombinant protein (MhCP) diluted in PBS at pH 4 was added to the spore suspension. The mixture was vortexed, and then mixed in a HulaMixer (Invitrogen, Waltham, MA) for 1 h at 4 °C. The binding mixture was centrifuged (5000×*g*, 2 min, 4 °C) and the pellet was washed two times with PBS buffer (pH 4). The washed pellet was resuspended in 100 µl of spore coat extraction buffer, incubated at 65 °C for 30 min to solubilise spore coat proteins, and one tenth of the above mixture was loaded onto a 12% SDS-PAGE gel^16^. Finally, the protein was confirmed by western blotting using anti-His antibodies (ThermoFisher, Canada).

### Animals and experimental design

The immunogenicity of recombinant protein MhCP was evaluated in a murine model. Balb/c 6–8-week-old, pathogen-free mice (Charles River Laboratories, Quebec, Canada) were housed in the Lethbridge Research and Development Centre (LeRDC) Vivarium adhering to procedures specified in the Canadian Council on Animal Care guidelines. The project was approved by the LeRDC Animal Care Committee (Animal Use Protocol # 1712) before commencement of the experiment and the current study was carried out in compliance with the ARRIVE guidelines. Mice were acclimatized to their cages for a week before commencement of the experiment. Mice were maintained on a Laboratory Rodent Diet 5001 (LabDiet, St. Louis, MO), and they were allowed to eat and drink ad libitum. They were monitored daily during the entire period of this experiment and a feeding and health log was maintained. A total of 40 mice were divided into the following four experimental groups (N = 10 mice per treatment): intramuscular (IM; free MhCP, positive control for reference of antigen immunogenicity); intragastric (IG; spore-bound MhCP); intranasal (IN; spore-bound MhCP); and naïve/control mice (Control; no MhCP used, negative control). In an initial study, spores were analyzed for potential to elicit immune responses in mice (N = 6) on day 42, after primary (day 0) and booster (day 21) IN vaccinations of only spores. No detectable IgG (serum) or IgA (BAL) against PlpE or NLKT were observed (data not shown). Therefore, an IN spore only group was not included in the study design.

### Immunisations and sample collection

To evaluate the immune response, mice were immunized through respective routes on day 0 followed by a booster immunization on day 21. A total of 5 µg of recombinant protein MhCP was administered per mice on each immunization day. For intranasal vaccination, spore-bound antigen (Spore-MhCP) was administered by pipette while mice were under light anaesthesia. Mice were restrained, holding them by the scruff, with thumb and index fingers and pressing the tail with the ring finger against the palm. A half-dose volume of the Spore-MhCP was delivered to each nostril using a micropipette fitted with a tip. For intramuscular immunization, free antigen MhCP mixed with Freund's incomplete adjuvant (Sigma-Aldrich, Canada) was inoculated into the thigh muscles of anaesthetized mice. The intragastric immunization was performed through oral gavage using a plastic feeding tube (Instech Laboratories, PA, USA) for gastric delivery of Spore-MhCP. Control treatment mice were not administered any antigen. Samples (blood, BAL, fecal pellets, and saliva) were collected on days 21 and 42. At each sampling point, 5 mice from each group were sacrificed under deep anaesthesia. Each animal was transferred into an individual sterile cage and kept there for 3–5 min to collect the freshly voided feces. Saliva was collected afterwards using a sterile oral swab (Isohelix, UK). For blood sampling, anaesthesia was induced with 4% isoflurane in an induction chamber and maintained with 2% isoflurane using a somnosuite anaesthesia mask (Kent Scientific Corporation, Torrington, CT). Upon opening of the abdominal cavity, blood was collected from the caudal vena cava under anesthesia and animals were then humanly euthanized by exsanguinating. Immediately after death, BAL was collected by inserting a catheter in the trachea of mice, through which PBS solution was instilled into the bronchioles. The infused liquid was then gently retracted for a maximum BAL fluid recovery. To determine the effectiveness of the MhCP and the immunogenicity from each vaccination route, each sample was analyzed quantitatively for specific IgG and IgA.

### Quantification of antigen-specific serum IgG

Antigen-specific antibody (IgG) responses in serum were quantified by enzyme-linked immunosorbent assay (ELISA). Purified recombinant antigens, PlpE and NLKT, were used as ligands independently to coat ELISA plates (Nunc-Immuno MaxiSorp) at concentrations of 50 ng/well, and plates were incubated at 4 °C overnight. Sera collected from mice were used as primary antibodies. Naïve mice sera were used as control. After being blocked with ELISA Blocking Solution (50 mM Tris, 0.14 M NaCl, 1% BSA, pH 8.0) for 1 h at 37 °C, serum samples were diluted (1:1600 dilution for intramuscular treatment mice, and 1:50 for other treatment mice) in sample diluent (50 mM Tris, 0.14 M NaCl, 1% BSA, 0.05% Tween 20), and 100 µL aliquots of each dilution were dispensed into wells of microtiter plates in duplicate. Plates were incubated for 1 h at room temperature. Horseradish peroxidase-conjugated anti-mouse IgG (Bethyl Laboratories Inc.) was used as secondary antibody. Following the addition of the secondary antibody, plates were incubated for 1 h at room temperature, and then developed with the substrate, TMB (3,3′,5,5′-tetramentylbenzidine; ThermoFisher, Canada). ELISA plates were washed five times with wash buffer. Reactions were stopped by ELISA stop solution (0.2 M H_2_SO_4_), and fluorescence was measured at 450 nm using a Microtiter Plate Reader (Synergy HTX Microplate Reader, BioTek). Concentrations were determined from a standard curve generated using a Mouse IgG ELISA Quantitation Set protocol (Cat. No. E90-131; Bethyl Laboratories Inc.). Known concentrations of Mouse Reference Serum (RS10-101-5; Bethyl Laboratories Inc.) was used.

### Quantification of secretory IgA (sIgA)

BAL, fecal, and oral swab samples were processed for IgA quantification as described by Hoang et al. (2008)^47^. For BAL, samples were washed with PBS containing 0.1% (wt/vol) BSA and 1 mM of phenyl methyl sulphonyl fluoride (PMSF), followed by centrifugation at 17,000×*g* for 10 min. The resultant supernatant was used for IgA analysis. For fecal samples, freshly voided fecal materials were collected and frozen at − 80 °C until use. Approximately 0.1 g of feces was thawed in 400 µL PBS containing 1% BSA, 1 mM PMSF, 0.05% Tween 20, and 0.1% Triton X-100 with vortexing to disrupt particulate material. Samples were then centrifuged and supernatants were used for analysis as above. For saliva, oral swabs were incubated in PBS containing 0.1% BSA, 1 mM PMSF, and 0.05% Tween 20 for 1 h at room temperature. Samples were then centrifuged and supernatants were used for analysis. After pre-processing, the concentrations of mucosal secretory IgA (sIgA) were evaluated by ELISA as per the Mouse IgA ELISA Quantitation Set Protocol (Cat. No. E90-103; Bethyl Laboratories Inc.). Briefly, Nunc-Immuno MaxiSorp plates were coated with 50 ng/well of purified recombinant PlpE and NLKT, and incubated at 4 °C overnight. After being blocked with ELISA Blocking Solution (50 mM Tris, 0.14 M NaCl, 1% BSA, pH 8.0) for 1 h at room temperature, samples were diluted in sample diluent (50 mM Tris, 0.14 M NaCl, 1% BSA, 0.05% Tween 20), and aliquoted to individual microtitre plate wells in duplicate. Plates were incubated for 1 h at room temperature. After a wash step and the addition of HRP Conjugated anti-Mouse IgA Detection Antibody (A90-103P; Bethyl Laboratories Inc.), plates were further incubated for 1 h at room temperature. Color development was performed with addition of the TMB substrate. Reactions were stopped by adding ELISA stop solution, and fluorescence was measured at 450 nm using the Microtiter Plate Reader.

### Complement-mediated serum bactericidal activity assay

Antibodies in mice sera collected on day 42 (N = 3 randomly selected sera from each treatment, measured in duplicate) were evaluated for their complement-dependent bactericidal activity against *M. haemolytica* using a serum bactericidal activity (SBA) assay as previously described^27^. Serum from a newborn colostrum-deprived calf was used as the external source of complement and was screened for minimal or no intrinsic bactericidal activity towards *M. haemolytica*. Mouse sera tested in complement-mediated SBA assay were heat-inactivated at 56 °C for 30 min, prior to use, to remove endogenous complement activity. For *M. haemolytica* cell preparation, a serotype 1 strain was grown in brain heart infusion (BHI) broth, pelleted by centrifugation, washed with PBS, and finally re-suspended in PBS. To remove the polysaccharide capsule and maximize the exposure of surface epitopes, cells were decapsulated by incubating at 41 °C with 100 rpm shaking for 1 h. The decapsulated cells were re-suspended to an optical density (600 nm) of 0.50 in PBS and 1:1000 dilution of this solution was used in the SBA assay. Heat-inactivated mice sera (25 µl), exogenous complement source (25 µl) and decapsulated *M. haemolytica* cells (25 µl) were mixed and spread onto plates at the onset of the experiment (T0) and after 30 min of incubation at 37 °C (T30). Colonies were enumerated after 16 h of incubation at 37 °C with 5% CO2. Finally, the percent killing was calculated as: [(T0 growth − T30 growth)/T0 growth] × 100% for each replicate. Only complement source without antibodies (mice serum) served as negative control in this SBA assay.

### Statistical analysis

Kruskal–Wallis test was performed to analyze the ELISA results. Significance was tested against the control by Kruskal–Wallis test with post-hoc Dunn’s multiple comparison test where p-value less than 0.05 was considered statistically significant. One-way ANOVA were performed to analyze the SBA assay results. Significance was tested against the control by one-way ANOVA with Tukey’s posttest where p-value less than 0.05 was considered statistically significant. All analyses were performed using Microsoft Excel 2010 and GraphPad Prism version 9.

### Supplementary Information

Supplementary Information.

## Data Availability

The datasets used in the current study are available from the corresponding author upon reasonable request.
