# Supplementary material for: Development of a spore-based mucosal vaccine against the bovine respiratory pathogen Mannheimia haemolytica
Source: Sci Rep. 2023 Aug 10;13:12981. doi: 10.1038/s41598-023-29732-4 (PMC10415371; doi:10.1038/s41598-023-29732-4)
Supplement: Supplementary file 1 — Supplementary Information. [file 41598_2023_29732_MOESM1_ESM.pdf]

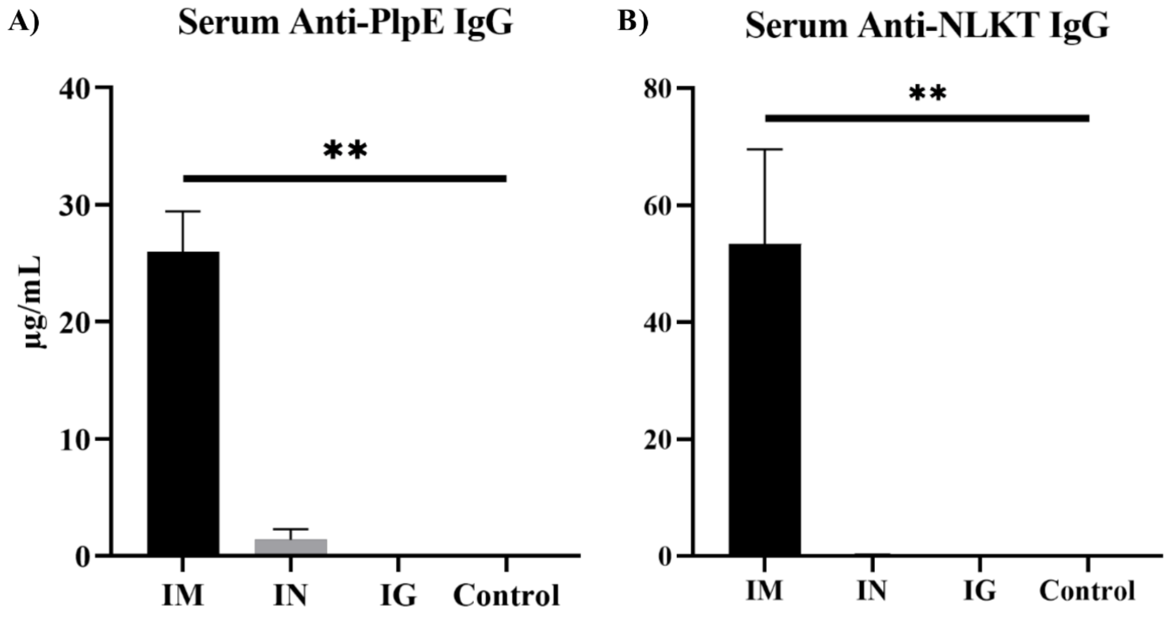

Supplemental Figure S1: Antigen-specific serum IgG antibody responses measured by enzyme linked immunosorbent assay (ELISA) from samples collected on day 21. ELISA plates coated with either recombinant PlpE or NLKT were used to measure anti-PlpE and anti-NLKT antibodies in mice sera from day 21. Immune responses from four experimental groups: Intramuscular (IM), Intranasal (IN), Intragastric (IG) and Naïve/control mice (Control) were compared. (A) Levels of serum IgG specific to PlpE (B) Levels of serum IgG specific to NLKT. Results are expressed as mean ± SEM. Significance was tested against the control by Kruskal-Wallis test with post-hoc Dunn's multiple comparison test, $^{**}p < 0.01$.

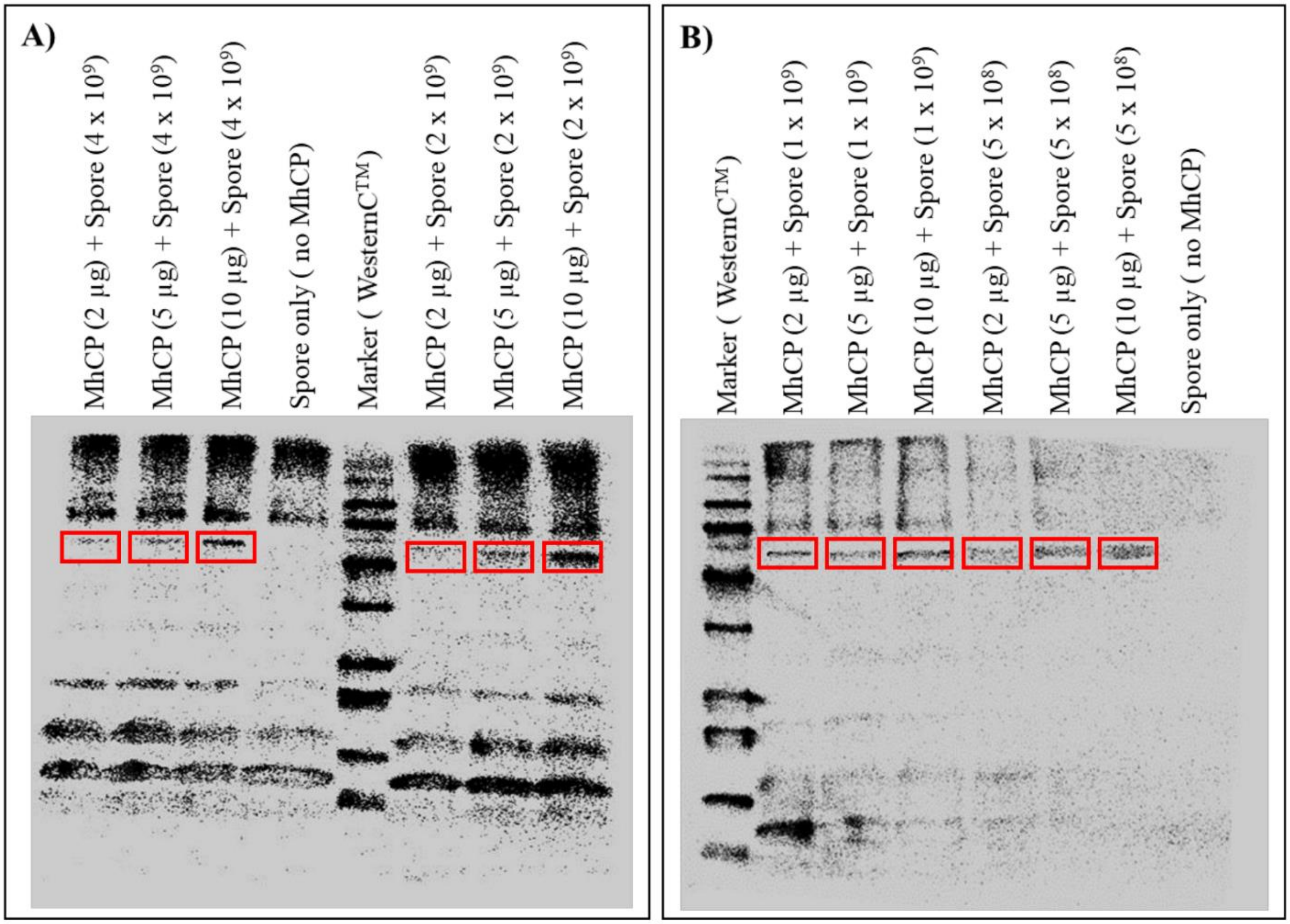

Supplemental Figure S2: Optimization of spore-to-antigen ratio. Chimeric protein, MhCP, of varying amounts (2 µg, 5 µg, 10 µg) were mixed with a range of *Bacillus subtilis* spores (4 x 10^9 to 5 x 10^8) in phosphate buffered saline (PBS), incubated for 1 h, washed, and resuspended in PBS. The supernatant was removed, the pellet containing spore coat antigens was washed and extracted by incubating in extraction buffer for 30 min at 65°C. Quantities of MhCP were determined by Western blotting. Spore only lane displays the corresponding blot generated from spores that underwent the entire process without the addition of protein MhCP. Outlined red boxes indicate the cropped regions denoted in Figure 2.

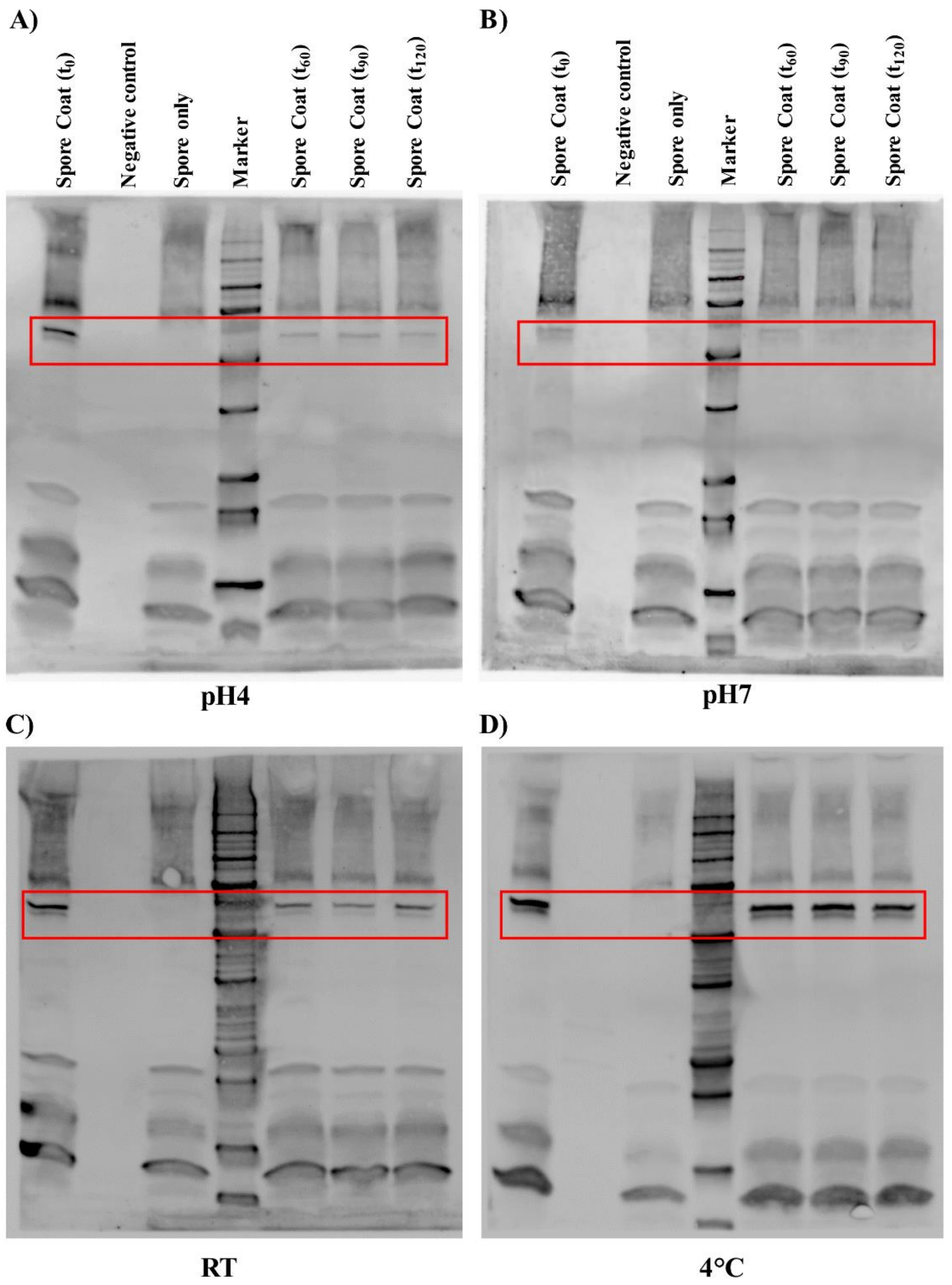

Supplemental Figure S3: Adsorption of chimeric protein MhCP to *Bacillus subtilis* spores. MhCP adsorbtion to spores was evaluated at different pHs (A, B) and temperatures (C, D). 10 µg of MhCP was mixed with 2 x 10^9 spores in phosphate buffered saline (PBS) at pH 4 (A) and PBS at pH 7 (B), incubated 1 h at room temperature (RT). The binding mixture was centrifuged and the pellet was washed two times with PBS. The washed pellet was next resuspended in 200 µl of PBS at their respective pH. At indicated time points (60, 90 and 120 min), the spore suspensions were centrifuged. Pellets were resuspended in 100 µl of spore coat extraction buffer, incubated at 65 °C for 30 min to remove spore coat proteins from the spores. Using a one in ten dilution of the extraction, western blotting of size-fractionated proteins was used for detection. Equivalent amounts of spores and protein were used at pH 4 (C, D). After the initial 1 h incubation at RT, the binding mixture was incubated at 4 °C (C), whereas all reactions and incubations were performed at 4 °C (D). The negative control lane displays the corresponding blot that underwent the entire process without the addition of any spores or protein. Outlined red boxes indicate the lanes denoted in Figure 3.

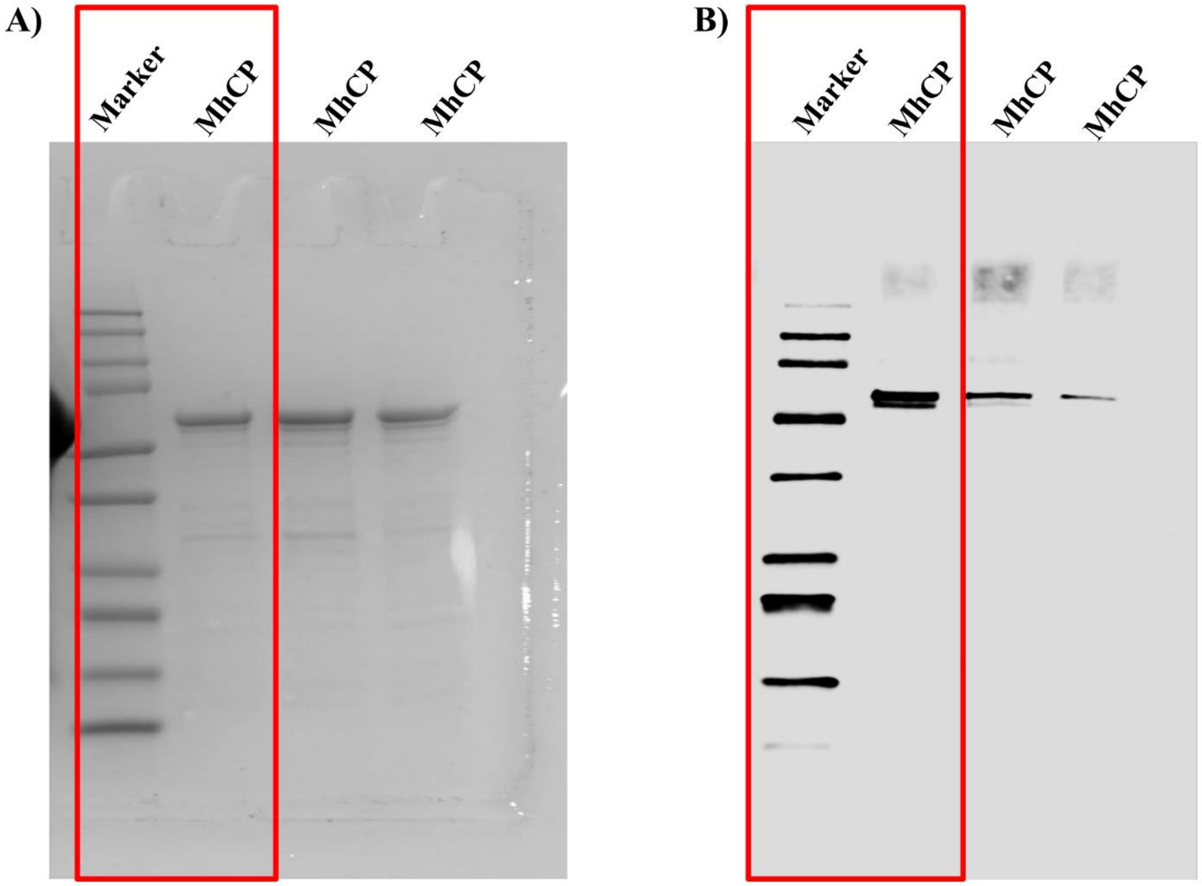

Supplemental Figure S4: Purified recombinant chimeric protein MhCP. A) Sodium dodecyl sulfate-polyacrylamide gel electrophoresis (SDS-PAGE) demonstrated the purified recombinant chimeric protein MhCP, stained with Coomassie Brilliant Blue. B) Identity of the MhCP protein was confirmed by western blots using anti-His antibodies. Outlined red boxes indicate the cropped regions denoted in Figure 1B and 1C.
